# Supplementary material for: Local assembly of long reads enables phylogenomics of transposable elements in a polyploid cell line
Source: Nucleic Acids Res. 2022 Sep 26;50(21):e124. doi: 10.1093/nar/gkac794 (PMC9757076; doi:10.1093/nar/gkac794)
Supplement: gkac794_Supplemental_Files [file gkac794_supplemental_files.zip › supplemental_dias_et_al.pdf]

Supplementary material for

Local assembly of long reads enables phylogenomics of transposable elements in a polyploid cell line

Shunhua Han[*,1], Guilherme B. Dias[*,†,1], Preston J. Basting[*], Raghuvir Viswanatha[‡]
Norbert Perrimon[‡,§] & Casey M. Bergman[*,†,2]

[*] Institute of Bioinformatics, University of Georgia, 120 E. Green St., Athens, GA, USA.
[†] Department of Genetics, University of Georgia, 120 E. Green St., Athens, GA, USA.
[‡] Department of Genetics, Harvard Medical School, 77 Avenue Louis Pasteur, Boston, MA, USA.
[§] Howard Hughes Medical Institute, Boston, MA, USA.

[1] These authors contributed equally to this work.
[2] Author for correspondence: cbergman@uga.edu.

1 Supplementary Text

1.1 Evaluation of TELR non-reference TE insertion coordinate and family predictions

We generated synthetic datasets using reads simulated from the ISO1 (dm6) [1] and A4 (GCA_003401745.1) [2] genome assemblies to evaluate TELR on predicting non-reference TE insertion under different ploidy, zygosity and coverage settings. In principle, a good predictor should be able to accurately predict "non-reference" insertions that are present in genome 1 (e.g., ISO1) but absent from genome 2 (e.g., A4) using reads simulated from genome 1 mapped to genome 2. Synthetic datasets under different settings were created as follows: 1) We simulated Pacbio reads from ISO1 only to model diploid homozygous insertions. 2) We simulated and combined Pacbio reads from both ISO1 and A4 with equal coverages to model diploid heterozygous insertions. 3) We simulated and combined Pacbio reads ISO1 and A4 with 1:3, 2:2, 3:1, and 4:0 ratio to model tetraploid simplex, duplex, triplex and quadruplex insertions, respectively (Table S3) [3]. The simulations were conducted using pbsim2 (v2.0.1; P6C4 HMM model) [4] under 50X, 100X, 150X, and 200X coverages for all ploidy, zygosity, and coverage settings. The synthetic datasets were used as input to TELR to detect non-reference TE insertions (v0.2; revision bb90a5a; options: –assembler wtdbg2 –polisher flye -p 1). The A4 assembly was used as the reference genome and the v10.2 of the curated library of *D. melanogaster* canonical TE sequences (`https://github.com/bergmanlab/drosophila-transposons`) were used for these analyses.

As ground truth for evaluating TELR performance, curated TE annotations from the release 6.38 version of *D. melanogaster* ISO1 genome (`http://ftp.flybase.net/releases/FB2021_01/dmel_r6.38/gff/dmel-all-r6.38.gff.gz`) were lifted over to A4 genome assembly. We excluded the highly abundant and degenerate *INE-1* family from this analysis since this family has been reported to be inactive in *Drosophila* for millions of years [5, 6]. After excluding *INE-1* insertions, TE insertions in low recombination regions, TEs without TAF predictions, and TEs without flank alignment support for both sides, 1,163 curated TEs in ISO1 could be lifted over to A4 on the basis of their flanking regions. TELR predictions were considered true positives if the predicted TE insertion coordinates were within a 5bp window of a lifted over ISO1 TE annotation and if the predicted TE family was the same as the lifted over annotation. The final benchmark results for TELR applied to synthetic datasets are summarized in Table S3. The results suggested that TELR has high precision (≥95%) under all ploidy, zygosity and coverage settings. In contrast, TELR's recall was much lower, especially at low effective coverage levels. These results indicate that the non-reference TE insertion predictions made by TELR are highly accurate, however, the method has an appreciable false negative rate especially when the effective coverage is lower than 50X.

1.2 Evaluation of TE allele copy number estimation by TELR in diploid and tetraploid genomes

We estimated the TE allele copy number by multiplying TAF predicted by TELR and local copy number predicted by Control-FREEC [7]. The estimated TE allele copy number is then rounded into integer value. To evaluate the TE allele copy number estimation accuracy, we used synthetic datasets generated from ISO1 and A4 genome assemblies under different coverage, ploidy, and zygosity settings (see details in Section 1.1). Only non-*INE-1*, non-nested TE insertions from normal recombination regions, with flank alignment support for both sides, and for which the TAF could be calculated were included in this analysis. The benchmark results were summarized in Table S6 and Table S7 for diploid and tetraploid genomes, respectively. Our estimated TE allele copy number had over 99.4% precision for diploid genomes and over 90.5% precision for tetraploid genomes under all coverage levels.

1.3 Evaluate TELR on TE sequence quality

Ideally, TELR should produce local contig assemblies from ISO1 reads mapped to the A4 reference that can be perfectly aligned to the corresponding TE loci in the ISO1 reference genome assembly. To evaluate quality of local contigs and TE sequences assembled and polished by TELR, we used synthetic datasets generated from ISO1 and A4 genome assemblies under different coverage, ploidy, and zygosity settings (see details in Section 1.1). Only non-*INE-1*, non-nested TE insertions from normal recombination regions, with flank alignment support for both sides, and for which the TAF could be calculated were included in this analysis. For a given TELR run, each reported TE sequence plus 500bp flanks upstream and downstream of TE region in the local contig assembly (later referred to as "TELR TE locus") was aligned to the ISO1 genome assembly. All TELR TE loci can be uniquely aligned to ISO1 (Figure S13). Next, for each TE locus predicted by TELR that have a corresponding curated TE annotation, we compared the TELR TE sequence with corresponding TE sequence in ISO1 based on curated TE annotation.

The nucleotide identity between TELR TE sequences and corresponding TE sequences in ISO1 are summarized in Figure S14. Note that although all TELR TE loci can be aligned to ISO1, a subset of TELR TE loci don't have corresponding curated TE annotation (mean = 29.1, sd = 5.14 for number of unmatched TELR TE loci across all TELR runs), which can be explained by missing data in the curated TE annotation.

2 Supplementary Tables

Table S1: **Statistics for S2R+ genome assemblies.** FALCON-Unzip_p = primary contigs; FALCON-Unzip_h = haplotigs; FALCON-Unzip_ph = primary + haplotigs.

	Canu	Falcon-Unzip_p	Falcon-Unzip_h	Falcon-Unzip_ph	wtdbg2	Flye	Supernova	SPAdes
Assembly size (bp)	288,352,585	166,182,448	43,221,666	209,404,114	146,747,034	144,559,338	136,942,585	138,619,786
Contig count	4,388	599	1,132	1,731	1,516	1,258	6,083	144,828
Contig N50 (bp)	133,624	711,969	41,606	494,779	448,074	478,824	78,941	42,786
Scaffold count	4,388	599	1,132	1,731	1,516	1,243	3,687	143,874
Scaffold N50 (bp)	133,624	711,969	41,606	494,779	448,074	484,692	874,516	54,714
GC content (%)	41.49	41.75	41.75	41.75	41.62	41.88	42.2	42.72
TEs (bp)	67,687,512	37,394,886	9,297,197	46,692,083	25,542,314	25,822,786	16,928,725	9,923,944
TEs (%)	23.47	22.50	21.51	22.30	17.41	17.86	12.36	7.16
BUSCO (%)								
Complete	99.6	97.1	25.6	98.5	95.1	99.0	98.7	98.7
Single-copy	63.1	91.4	25.1	76.3	93.8	97.6	98.0	98.5
Duplicated	36.5	5.7	0.5	22.2	1.3	1.4	0.7	0.2
Fragmented	0.1	0.8	2.4	0.2	0.4	0.3	0.3	0.6
Missing	0.3	2.1	72.0	1.3	4.5	0.7	1.0	0.7

Table S2: **Statistics for A4 genome assemblies.** FALCON-Unzip_p = primary contigs; FALCON-Unzip_h = haplotigs; FALCON-Unzip_ph = primary + haplotigs.

	Canu	Falcon-Unzip_p	Falcon-Unzip_h	Falcon-Unzip_ph	wtdbg2	Flye	Supernova	SPAdes
Assembly size (bp)	141,737,450	141,292,095	13,036,468	154,328,563	137,203,985	135,706,987	126,833,864	135,820,998
Contig count	181	107	289	396	431	234	3115	119370
Contig N50 (bp)	21,369,333	20,430,351	47,610	16,510,272	13,821,893	5,389,879	182,420	74,783
Scaffold count	181	107	289	396	431	229	1,818	118,640
Scaffold N50 (bp)	21,369,333	20,430,351	47,610	16,510,272	13,821,893	6,405,908	5,040,789	98,019
GC content (%)	42.07	42.14	41.89	42.12	41.83	42.11	42.27	42.33
TEs (bp)	21580457	22,804,030	1,803,901	24,607,939	20,832,191	18,259,520	11,339,728	9,393,794
TEs (%)	15.23	16.14	13.84	15.95	15.18	13.46	8.94	6.92
BUSCO (%)								
Complete	99.4	99.2	8.7	99.4	94.6	99.5	99.2	99.2
Single-copy	98.5	98.7	8.6	92.5	94.1	99.1	98.9	99.1
Duplicated	0.9	0.5	0.1	6.9	0.5	0.4	0.3	0.1
Fragmented	0.2	0.2	0.8	0.2	0.2	0.2	0.3	0.3
Missing	0.4	0.6	90.5	0.4	5.2	0.3	0.5	0.5

Table S3: **Evaluation of TELR non-reference TE prediction performance on simulated genomes.** Non-reference TE insertion predictions made by TELR on simulated datasets mapped to the A4 genome assembly as reference were evaluated against curated TE annotations in ISO1 lifted over to A4 coordinates (see Section 1.1 for details). "Zygosity" was simulated by controlling the ratio of simulated long reads generated using the ISO1 and A4 reference assemblies (see details in Section 1.1). "#TP" and "#FP" represent the number of true positive predictions that do and false positive predictions that don't match curated TE annotations in ISO1 lifted over to A4 coordinates, respectively. "#FN" represents the number of false negative curated TE annotations in ISO1 lifted over to A4 coordinates that were not predicted by TELR. "Precision" represents the percent of true positive predictions divided by total number of predictions made by TELR. "Recall" represents the percent of true positive predictions divided by total number of curated TE annotations in ISO1 lifted over to A4 coordinates. Only non-*INE-1*, non-nested TE insertions from normal recombination regions, with flanking alignment support for both sides, and for which the TAF could be calculated were included in this analysis.

Ploidy	Zygosity	Coverage	#Predictions	#TP	#FP	#FN	Precision	Recall	Predictions with TSD
diploid	homozygous	50	461	451	10	166	97.8%	73.1%	48.8%
diploid	homozygous	100	465	456	9	161	98.1%	73.9%	51.1%
diploid	homozygous	150	494	483	11	134	97.8%	78.3%	51.2%
diploid	homozygous	200	485	474	11	143	97.7%	76.8%	51.3%
diploid	heterozygous	50	374	369	5	248	98.7%	59.8%	49.7%
diploid	heterozygous	100	448	440	8	177	98.2%	71.3%	52.0%
diploid	heterozygous	150	470	460	10	157	97.9%	74.6%	50.5%
diploid	heterozygous	200	478	470	8	147	98.3%	76.2%	50.0%
tetraploid	simplex	50	105	105	0	512	100.0%	17.0%	46.7%
tetraploid	simplex	100	365	360	5	257	98.6%	58.3%	49.5%
tetraploid	simplex	150	425	415	10	202	97.6%	67.3%	49.1%
tetraploid	simplex	200	451	441	10	176	97.8%	71.5%	46.2%
tetraploid	duplex	50	364	356	8	261	97.8%	57.7%	47.3%
tetraploid	duplex	100	457	449	8	168	98.2%	72.8%	51.1%
tetraploid	duplex	150	470	460	10	157	97.9%	74.6%	47.6%
tetraploid	duplex	200	472	464	8	153	98.3%	75.2%	51.0%
tetraploid	triplex	50	446	438	8	179	98.2%	71.0%	44.6%
tetraploid	triplex	100	474	465	9	152	98.1%	75.4%	48.8%
tetraploid	triplex	150	480	472	8	145	98.3%	76.5%	53.1%
tetraploid	triplex	200	483	473	10	144	97.9%	76.7%	50.7%
tetraploid	quadruplex	50	465	456	9	161	98.1%	73.9%	52.1%
tetraploid	quadruplex	100	481	470	11	147	97.7%	76.2%	51.2%
tetraploid	quadruplex	150	490	481	9	136	98.2%	78.0%	55.0%
tetraploid	quadruplex	200	483	473	10	144	97.9%	76.7%	49.6%

Table S4: **Proportion of non-reference TEs with TSDs predicted by TELR in S2R+ and DSPR strains.** Overall numbers of non-reference TE predictions are shown in Fig. S5.

Sample	Predictions with TSD
S2R+	54.9%
A1	49.3%
A2	47.8%
A3	50.1%
A4	54.2%
A5	53.2%
A6	53.9%
A7	53.0%
AB8	46.7%
B1	48.3%
B2	52.0%
B3	48.1%
B4	49.5%
B6	52.5%

Table S5: **Number of non-reference TEs shared between A7 and DSPR strains.** BEDTools (v2.29.0) [8] was used to investigate the possibility of contamination in the A7 sample with DNA from another strain by intersecting A7 TELR predictions with all other DSPR strains. "#Overlaps" represents the number of non-reference TEs shared between A7 and each of the DSPR strain. Only non-*INE-1*, non-nested TE insertions from normal recombination regions, with flank alignment support for both sides, and for which the TAF could be calculated were included in this analysis.

Strain	#Overlaps
A1	9
A2	12
A3	7
A4	5
A5	16
A5	8
A7	660
AB8	8
B1	13
B2	7
B3	145
B4	10
B6	17

Table S6: **TELR performance for intra-sample TE allele copy number classification on diploid genomes.** TELR predictions on synthetic data from ISO1 and A4 genome assemblies were used as input for the classifier. "Zygosity" was simulated by controlling the ratio of simulated reads generated from ISO1 and A4 (see details in Section 1.1). "Precision" represents the proportion of TE allele copy number being correctly classified. Only non-*INE-1*, non-nested TE insertions from normal recombination regions, with flank alignment support for both sides, and for which the TAF could be calculated were included in this analysis.

Ploidy	Zygosity	Coverage	#Total Predictions	#TE Copy=1	#TE Copy=2	#Unclassified	Precision
diploid	homozygous	50	461	0	461	0	100.0%
diploid	homozygous	100	465	1	464	0	99.8%
diploid	homozygous	150	494	2	492	0	99.6%
diploid	homozygous	200	485	1	484	0	99.8%
diploid	heterozygous	50	374	372	2	0	99.5%
diploid	heterozygous	100	448	447	1	0	99.8%
diploid	heterozygous	150	470	469	1	0	99.8%
diploid	heterozygous	200	478	475	3	0	99.4%

Table S7: **TELR performance for intra-sample TE allele copy number classification on tetraploid genomes.** TELR predictions on synthetic data from ISO1 and A4 genome assemblies were used as input for the classifier. "Zygosity" was simulated by controlling the ratio of simulated reads generated from ISO1 and A4 (see details in Section 1.1). "Precision" represents the proportion of TE allele copy number being correctly classified. Only non-*INE-1*, non-nested TE insertions from normal recombination regions, with flank alignment support for both sides, and for which the TAF could be calculated were included in this analysis.

Ploidy	Zygosity	Coverage	#Total Predictions	#TE Copy=1	#TE Copy=2	#TE Copy=3	#TE Copy=4	Precision
tetraploid	simplex	50	105	95	10	0	0	90.5%
tetraploid	simplex	100	365	359	6	0	0	98.4%
tetraploid	simplex	150	425	419	5	0	1	98.6%
tetraploid	simplex	200	451	444	6	0	1	98.4%
tetraploid	duplex	50	364	9	346	8	1	95.1%
tetraploid	duplex	100	457	9	442	6	0	96.7%
tetraploid	duplex	150	470	0	463	5	2	98.5%
tetraploid	duplex	200	472	2	466	3	1	98.7%
tetraploid	triplex	50	446	0	14	416	16	93.3%
tetraploid	triplex	100	474	0	15	454	5	95.8%
tetraploid	triplex	150	480	0	5	471	4	98.1%
tetraploid	triplex	200	483	0	3	477	3	98.8%
tetraploid	quadruplex	50	465	0	0	0	465	100.0%
tetraploid	quadruplex	100	481	0	1	0	480	99.8%
tetraploid	quadruplex	150	490	0	1	0	489	99.8%
tetraploid	quadruplex	200	483	1	1	0	481	99.6%

Table S8: **Feature comparison between long-read non-reference TE detection methods.** *LoRTE does not predict genotypes but may flag TEs as "Possible polymorphism" if there is conflicting evidence regarding the presence/absence of a given insertion. This indicates the insertion is heterozygous or potentially a polymorphism if multiple individuals were pooled together for sequencing [9].

	TELR	LoRTE	PALMER	TLDR	xTea	rMETL	nanotei
PubMed ID	-	28405230	31853540	33186547	34158502	30759188	34961152
Species-agnostic	Yes	Yes	Yes	Yes	No	Yes	No
Predicts TSD	Yes	No	Yes	Yes	Yes	No	No
TE sequence	Polished local assembly	Representative raw read	Representative raw read	Consensus sequence	Unpolished local assembly	Yes	No
Estimates TAF	Yes	No	No	No	No	No	No
Predicts genotype	Yes	Polymorphic TE (*)	No	No	No	Yes	No
Available in Bioconda	Yes	No	No	No	Yes	Yes	No

3 Supplementary Figures

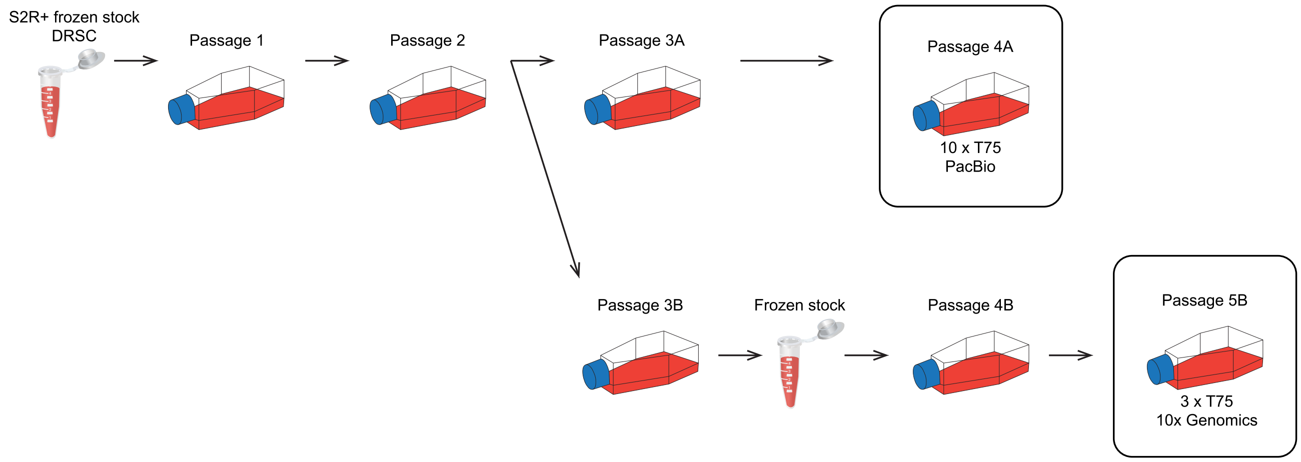

Figure S1: **Provenance of the S2R+ cell line samples used in this study.** Cells were harvested from passages 4A and 5B for PacBio and 10x Genomics linked-read DNA sequencing, respectively.

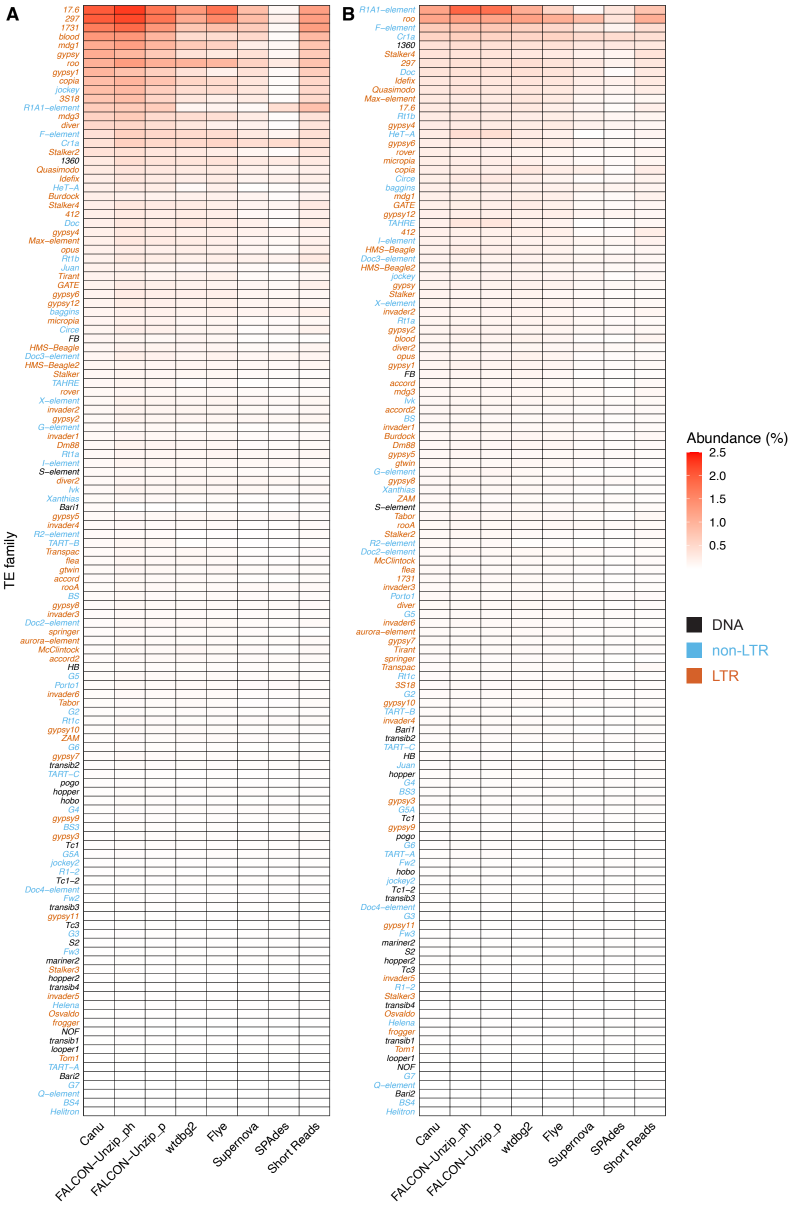

Figure S2: **Estimated TE abundance can vary substantially across whole-genome assembly methods.** TE abundance was estimated for different assemblies and directly from raw Illumina reads in S2R+ (A) and A4 (B) using RepeatMasker and the curated canonical *D. melanogaster* TE library. TE family names were colorized by TE type.

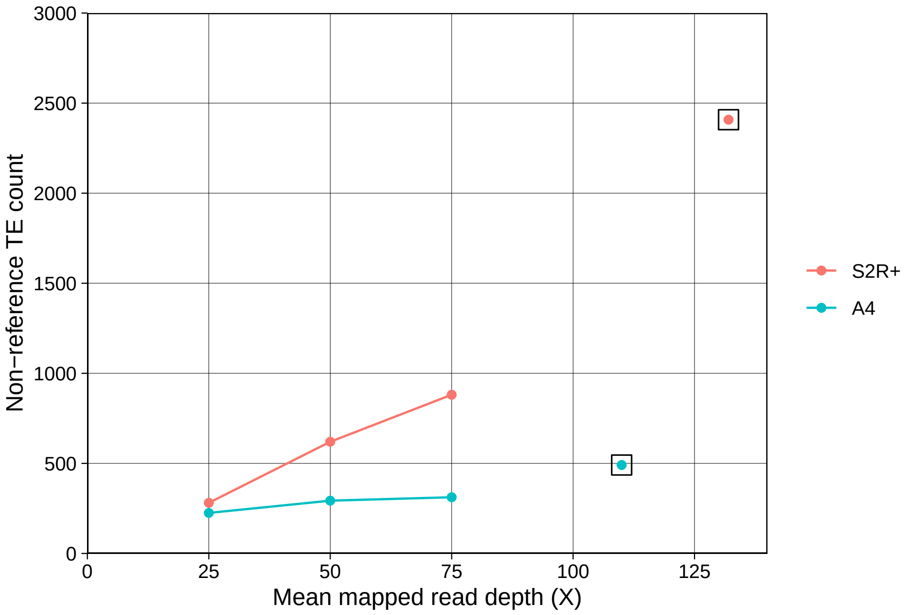

Figure S3: **Effect of sequencing coverage on the number of TELR predictions.** Comparison of the number of non-reference TEs predicted by TELR using length- and depth-normalized long-read datasets for S2R+ and A4. Reads from both datasets were split into 8kb segments and sub-sampled to achieve the desired mean mapped read depth. The boxed points indicate the number of TE predictions made by TELR when the full non-normalized data was supplied. Only non-*INE-1*, non-nested TE insertions from normal recombination regions, with flank alignment support for both sides, and for which the TAF could be calculated were included in this analysis.

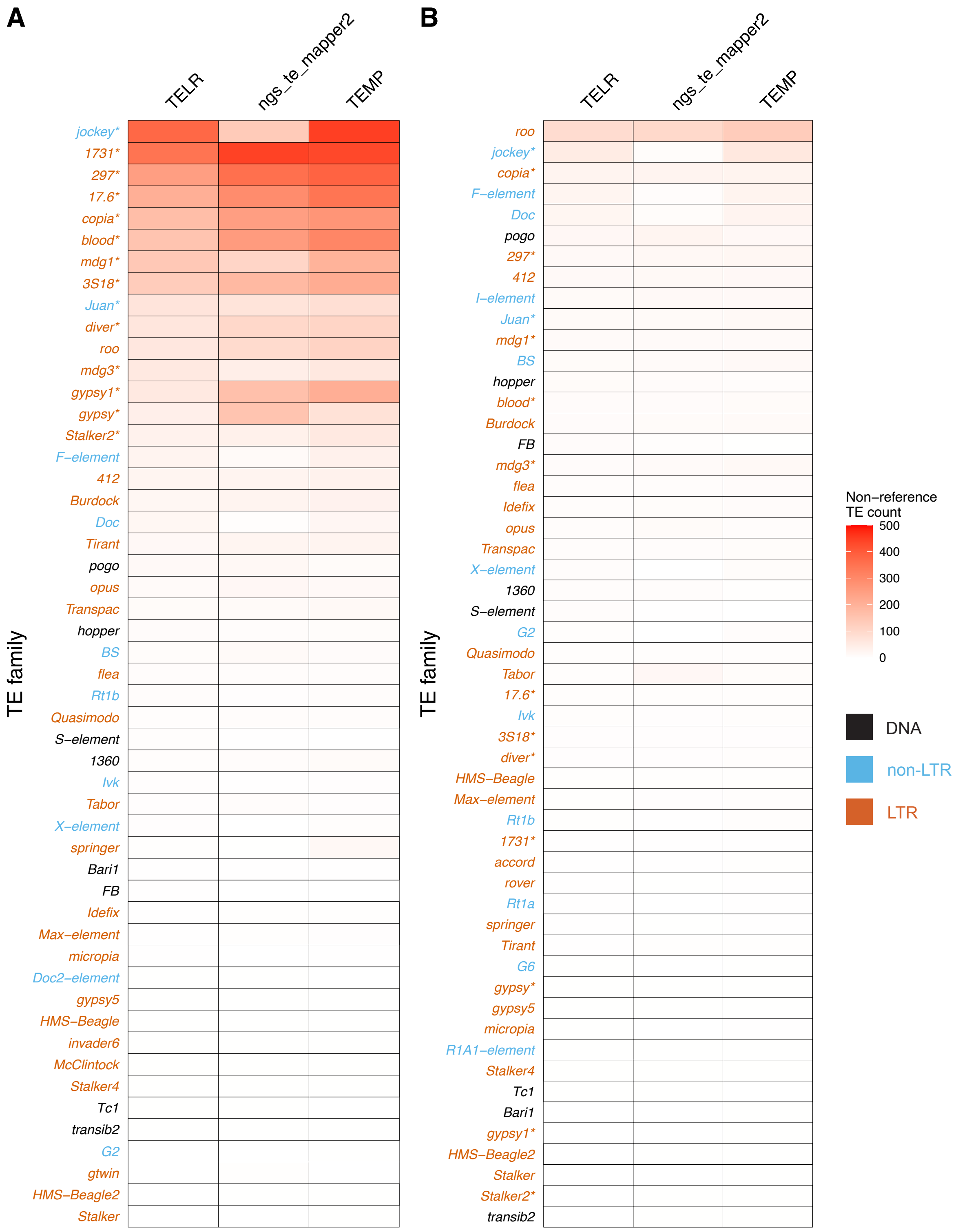

Figure S4: **Abundance of TE families detected by short-read methods in S2R+ and A4.** Heatmaps represent number of non-reference TEs separated by TE families for S2R+ (A) and A4 (B). TE family names were colorized by TE type. TE families enriched in S2R+ relative to A4 are indicated by asterisks. Only non-*INE-1*, non-nested TE insertions from normal recombination regions, with flank alignment support for both sides, and for which the TAF could be calculated were included in this analysis.

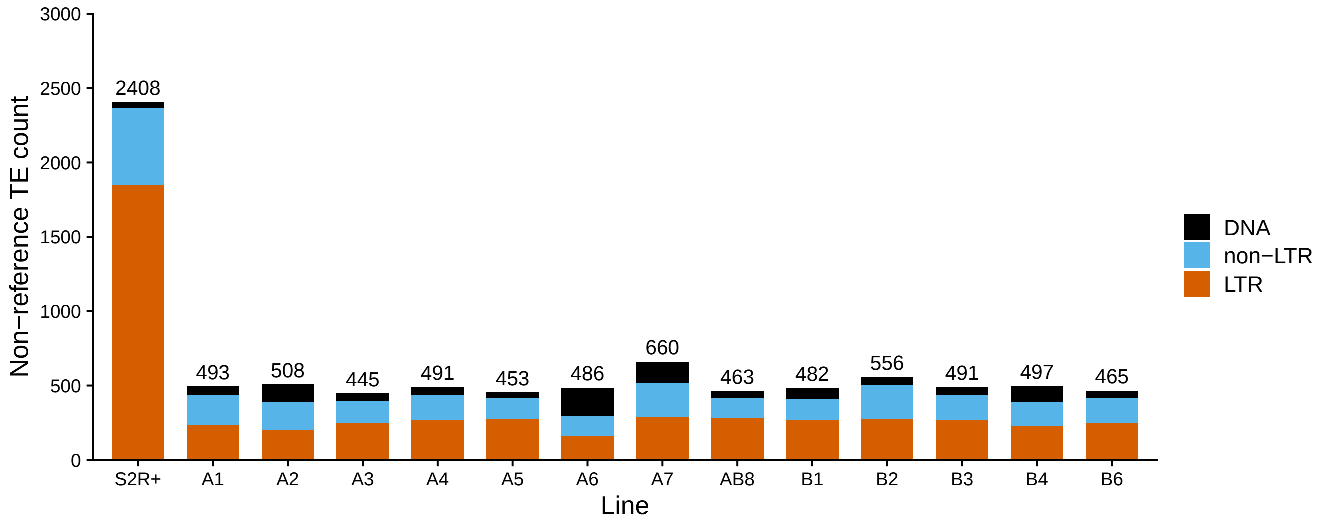

Figure S5: **Number of non-reference TE insertions in S2R+ and inbred fly stocks from the DSPR panel.** Number of non-reference TE insertions predicted by TELR for S2R+ and inbred stocks from geographically diverse natural fly populations [10]. The bars were colorized by TE type. Number on top of each bar represents total number of non-reference TE insertions for a given line. Only non-*INE-1*, non-nested TE insertions from normal recombination regions, with flank alignment support for both sides, and for which the TAF could be calculated were included in this analysis.

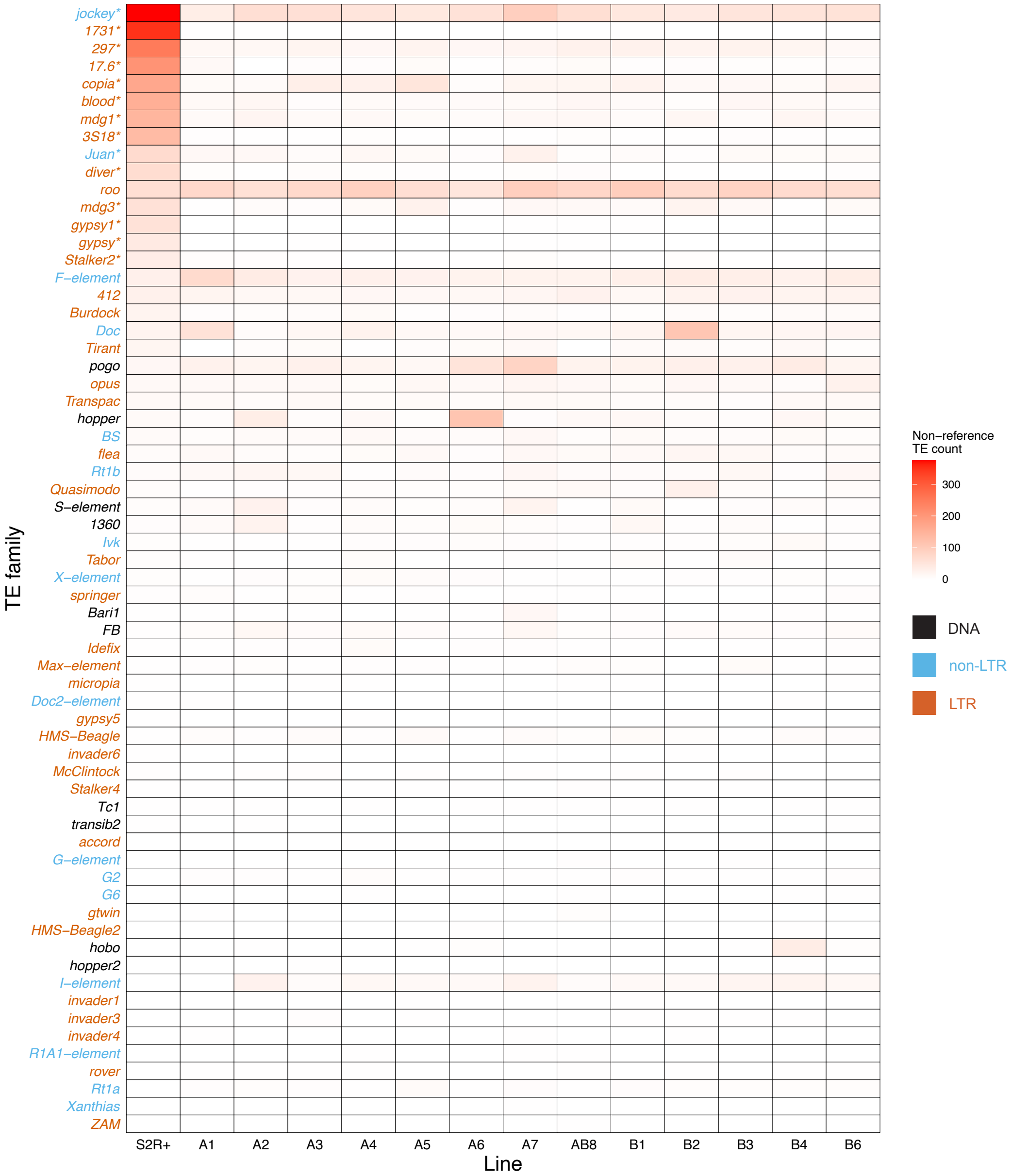

Figure S6: **Abundance of TE families detected by TELR in S2R+ and inbred fly strains in the DSPR panel.** Heatmap showing the copy number of non-reference insertions from multiple TE families in S2R+ and inbred fly strains from the *Drosophila* Synthetic Population Resource (DSPR) [11]. TE families are ordered according to their abundance in S2R+. TE family names were colorized by TE type. The 14 TE families enriched in S2R+ relative to A4 are indicated by asterisks. Only non-*INE-1*, non-nested TE insertions from normal recombination regions, with flank alignment support for both sides, and for which the TAF could be calculated were included in this analysis.

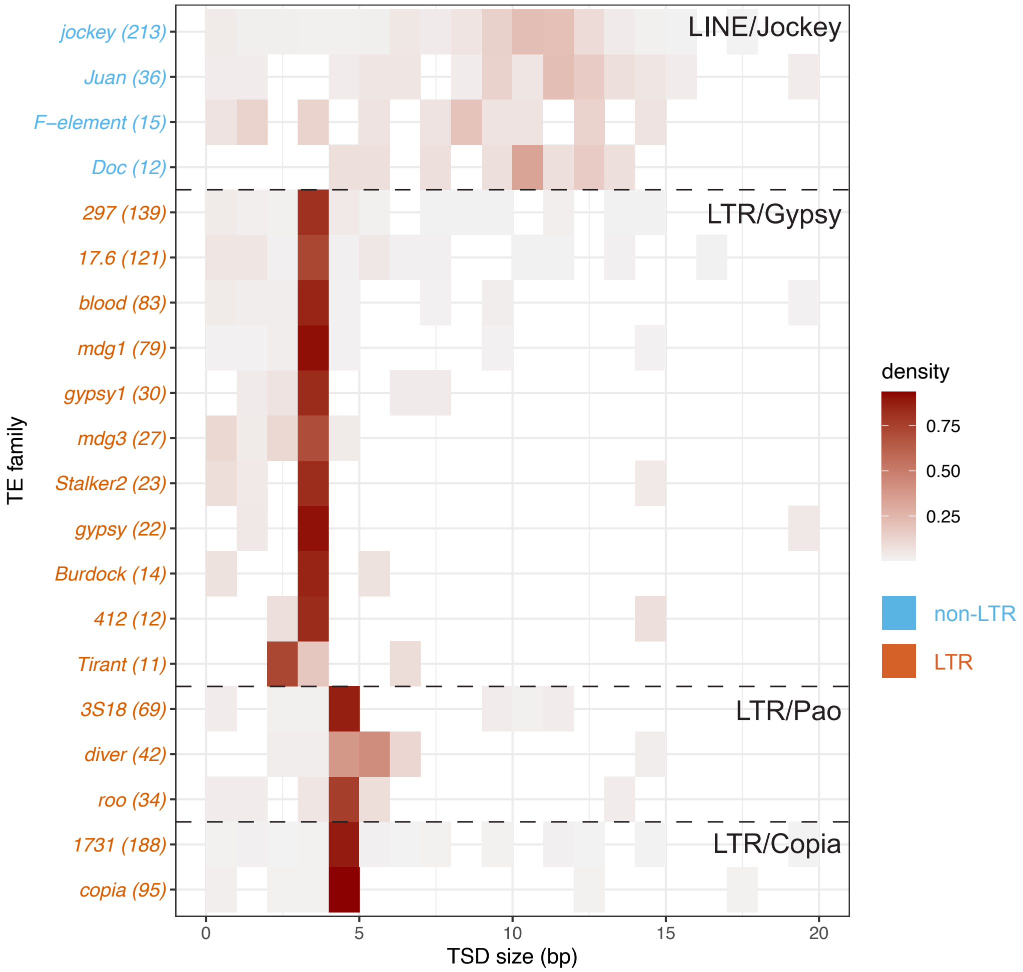

Figure S7: **Length distributions of TSDs predicted by TELR for abundant TE families in the S2R+ genome.** Heatmaps represent the distribution of TSD lengths within TE family. Distributions are shown for the 20 TE families with greater than ten non-reference TEs predicted by TELR in S2R+ that have TSD annotations.

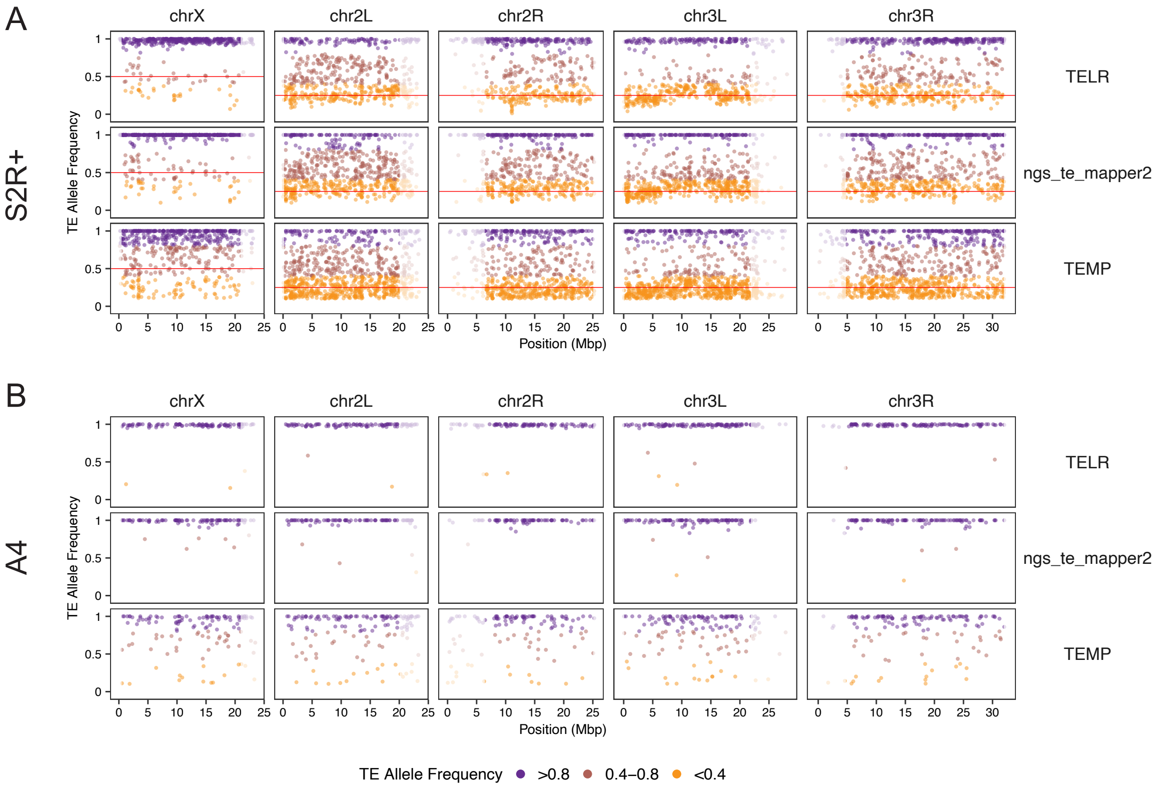

Figure S8: **Genome-wide TAF profiles for non-reference TE predictions made by TELR and short-read methods in S2R+ and A4.** Only non-*INE-1*, non-nested TE insertions with flank alignment support for both sides, and for which the TAF could be calculated were included in this analysis. Low recombination regions are indicated by higher transparency.

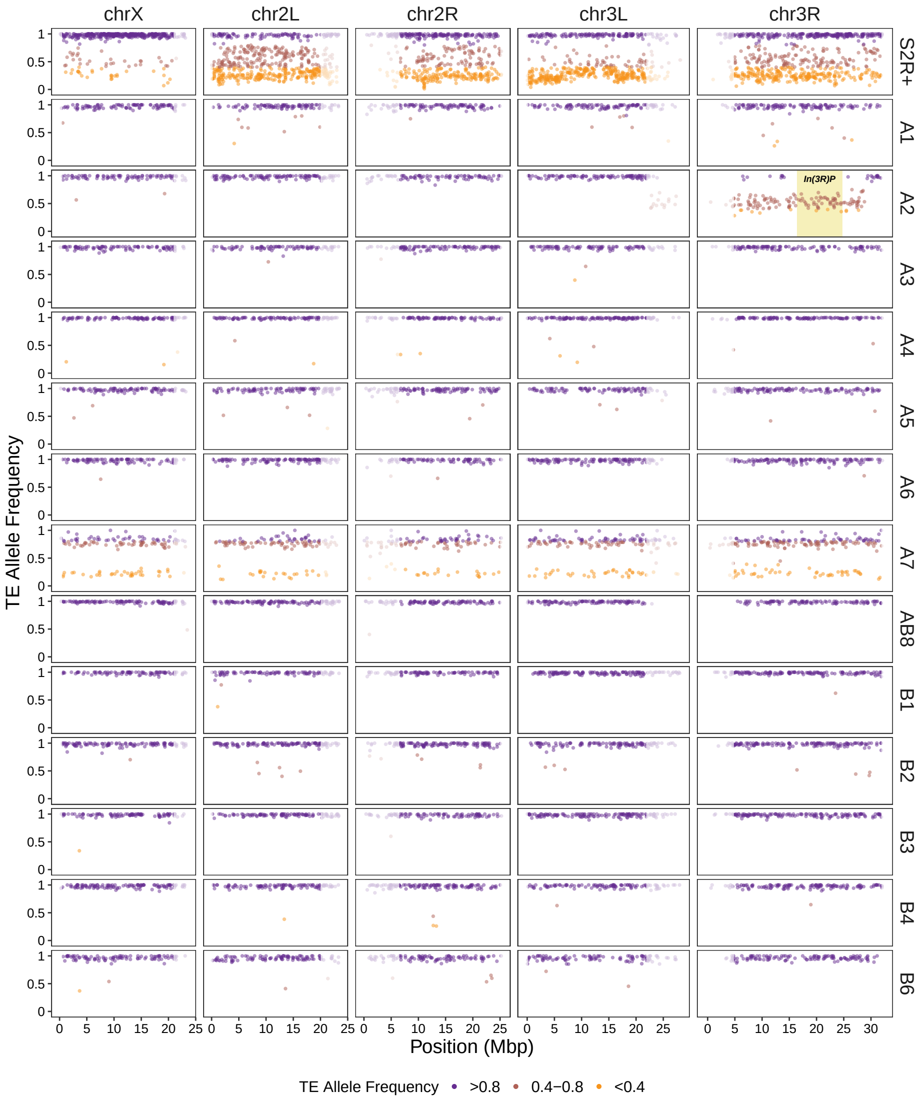

Figure S9: **Genome-wide TAF profiles for non-reference TE predictions made by TELR in S2R+ and inbred fly strains in the DSPR panel.** Strain A2 has a known heterozygous inversion, *In(3R)P*, that prevents the complete inbreeding in chr3R [11]. Only non-*INE-1*, non-nested TE insertions with flank alignment support for both sides, and for which the TAF could be calculated were included in this analysis. Low recombination regions are indicated by higher transparency.

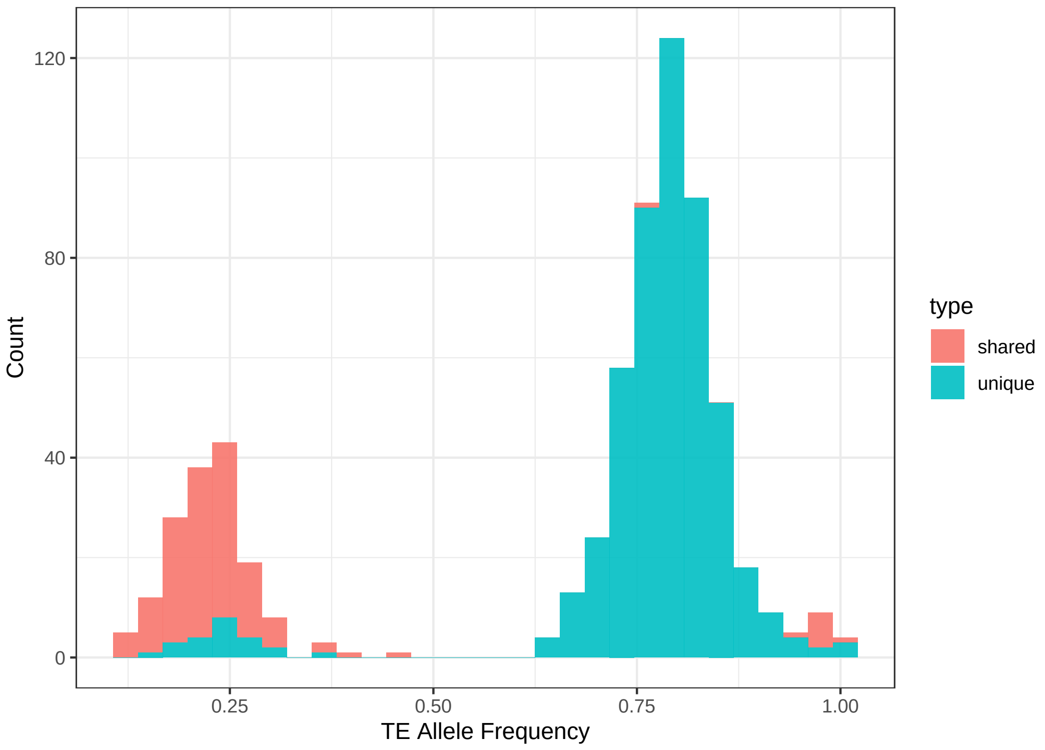

Figure S10: **TAF distribution of non-reference insertions in A7.** The histogram is colorized based on whether the TE insertion in strain A7 is shared with strain B3 or unique to A7. Only non-*INE-1*, non-nested TE insertions from normal recombination regions, with flank alignment support for both sides, and for which the TAF could be calculated were included in this analysis. TE insertions shared by A7 and B3 are enriched at TAFs of ∼0.25, consistent with ∼25% of sequences in the A7 sample being contaminated by sequences from strain B3.

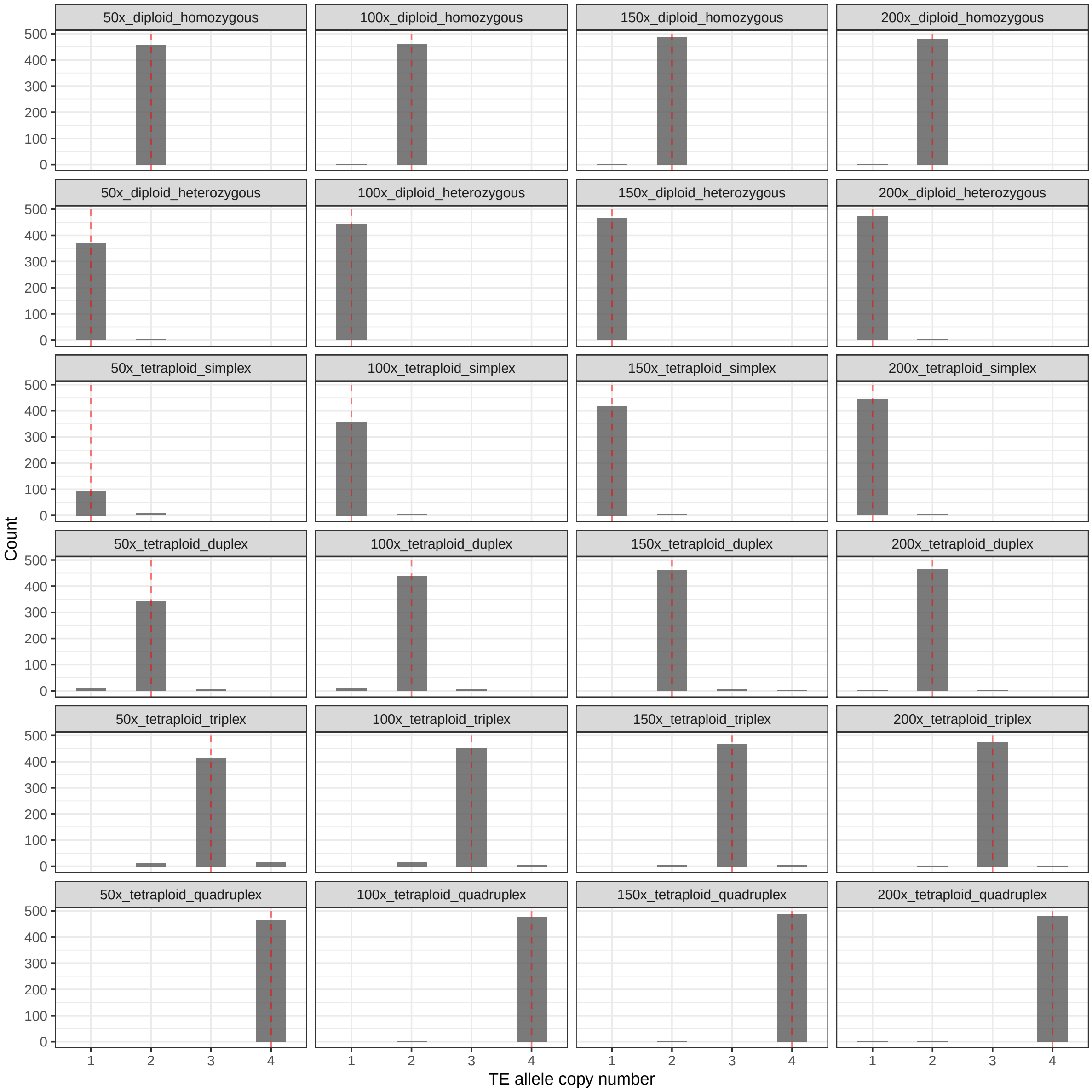

Figure S11: **TE allele copy number distribution using simulated long-read sequencing data.** Histograms of TE allele copy number on synthetic long-read dataset simulated under different ploidy, zygosity, and coverage settings. See section 1.1 on details about how simulated datasets were generated and how the TE allele copy number was estimated. The expected TE allele copy number under a given ploidy and zygosity setting is marked in dashed red line.

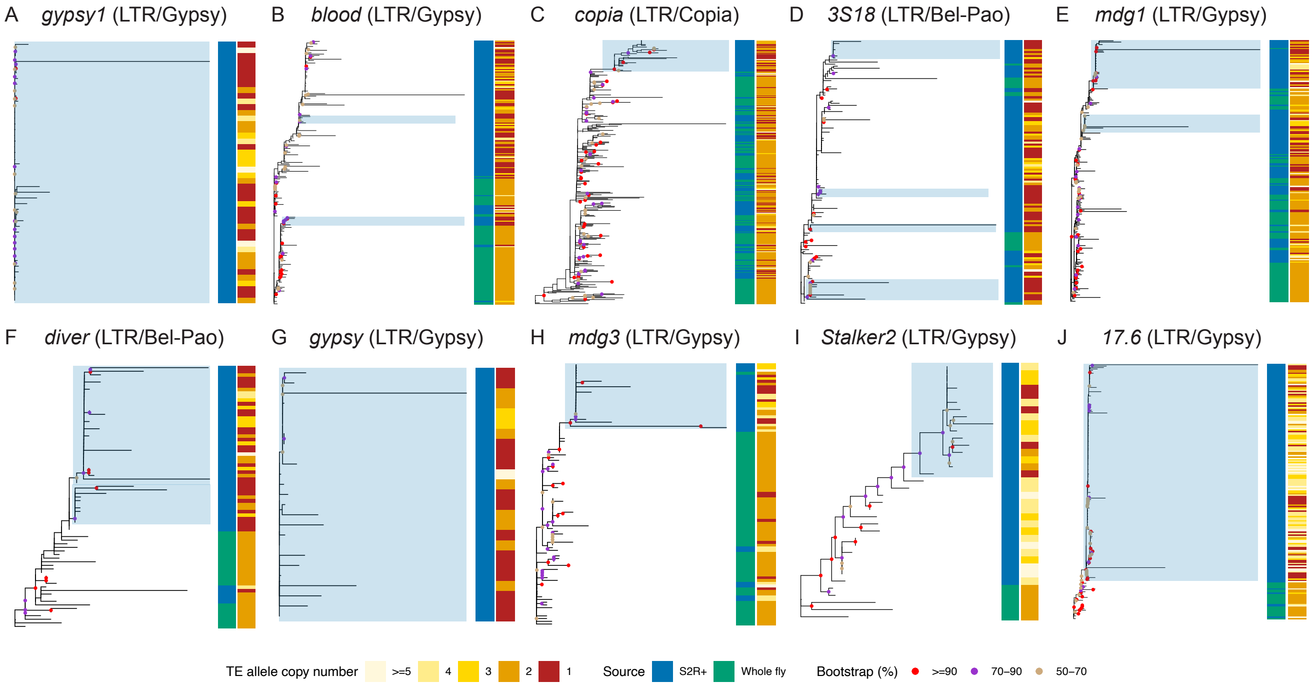

Figure S12: **Amplification of TE families in the S2R+ genome can be driven by one or more source lineage.** Non-reference TE insertion sequences from S2R+ and 11 inbred *Drosophila* fly strains were predicted and assembled by TELR. Full length TE sequences for each family were aligned using MAFFT (v7.487) [12]. The multiple sequence alignments were used as input in IQ-TREE (v2.1.4-beta) [13] to build unrooted trees for (A) *gypsy1*, (B) *blood*, (C) *copia*, (D) *3S18*, (E) *mdg1*, (F) *diver*, (G) *gypsy*, (H) *mdg3*, (I) *Stalker2* and (J) *17.6* elements using maximum likelihood approach. The sample source and TE allele copy number were annotated in the sidebars. Blue shading indicates TE expansion event in S2R+ from a single source lineage based on the following criteria: 1) all sequences should form a monophyletic clade; 2) the monophyletic clade should include at least three post-tetraploid cell-line-specific TE insertions; 3) the bootstrap support for the clade should be equal to or higher than 50%; and 4) the proportion of post-tetraploid cell-line-specific TE insertions (i.e. TE allele copy number equal to one) within the clade should be equal to or higher than 20%.

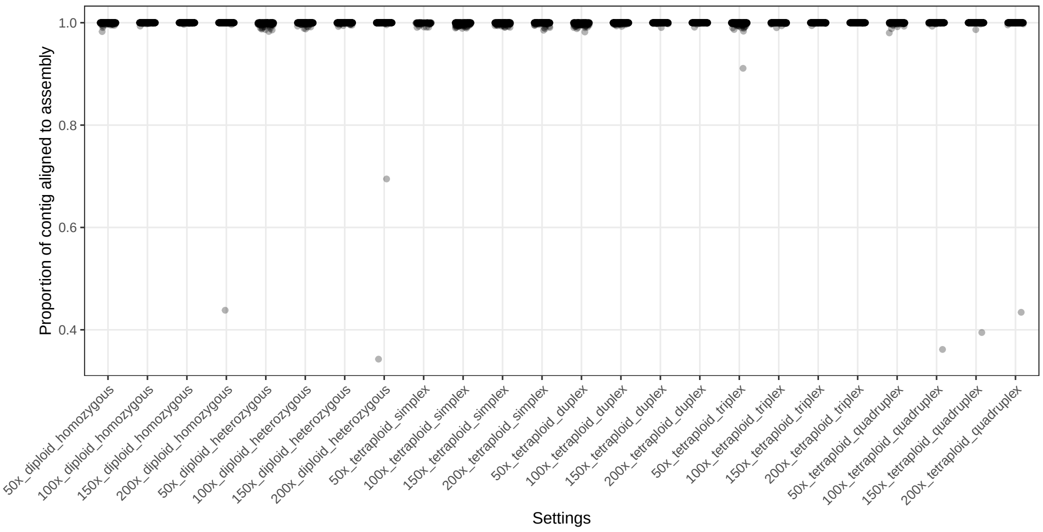

Figure S13: **Distribution on the proportion of TE loci assembled by TELR that can be aligned to the ISO1 reference genome assembly.** For a given TELR run using synthetic long-read sequencing data simulated under a specific coverage, ploidy, and zygosity setting, each TE sequence plus 500bp flanking sequences on 5' and 3' side of the TE locus in the local contig assembly made by TELR (referred to as "TELR TE locus") was aligned to the ISO1reference genome assembly. "Proportion of contig aligned to assembly" on the y-axis represents the proportion of each TELR TE locus that can be aligned to ISO1. See details in Section 1.3.

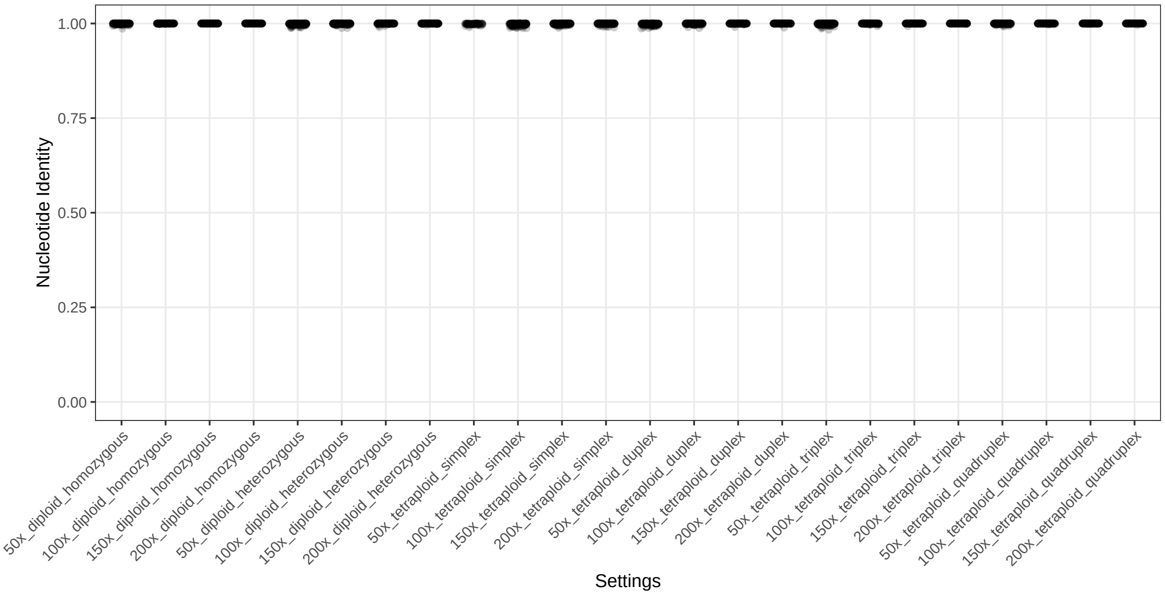

Figure S14: **Distribution of nucleotide identity between TELR TE sequences and corresponding TE sequences in the ISO1 reference genome in simulated long-read sequencing datasets.** For a given TELR run using synthetic long-read sequencing data simulated under a specific coverage, ploidy, and zygosity setting, each predicted TE sequence plus 500bp flanking sequences on 5' and 3' side of the TE locus in the local contig assembly (referred to as "TELR TE locus") was aligned to the ISO1 genome assembly. For each TE insertion predicted by TELR that can be matched with a corresponding curated TE annotation in ISO1 (FlyBase release 6.38), we aligned TELR TE sequence with TE sequences in ISO1 based on curated TE annotation using minimap2 [14] and calculated nucleotide identity. See details in Section 1.3.

References

[1] Hoskins, R. A., Carlson, J. W., Wan, K. H., Park, S., Mendez, I., Galle, S. E., Booth, B. W., Pfeiffer, B. D., George, R. A., Svirskas, R., Krzywinski, M., Schein, J., Accardo, M. C., Damia, E., Messina, G., Mendez-Lago, M., de Pablos, B., Demakova, O. V., Andreyeva, E. N., Boldyreva, L. V., Marra, M., Carvalho, A. B., Dimitri, P., Villasante, A., Zhimulev, I. F., Rubin, G. M., Karpen, G. H., and Celniker, S. E. (2015) The Release 6 reference sequence of the Drosophila melanogaster genome. *Genome Res,* **25**(3), 445–458.

[2] Chakraborty, M., VanKuren, N. W., Zhao, R., Zhang, X., Kalsow, S., and Emerson, J. J. (2018) Hidden genetic variation shapes the structure of functional elements in Drosophila. *Nat Genet,* **50**(1), 20–25.

[3] Blakeslee, A. F., Belling, J., and Farnham, M. E. (1923) Inheritance in tetraploid Daturas. *Botanical Gazette,* **76**(4), 329–373.

[4] Ono, Y., Asai, K., and Hamada, M. (2021) PBSIM2: a simulator for long-read sequencers with a novel generative model of quality scores. *Bioinformatics,* **37**(5), 589–595.

[5] Singh, N. D. and Petrov, D. A. (2004) Rapid sequence turnover at an intergenic locus in Drosophila. *Molecular Biology and Evolution,* **21**(4), 670–80.

[6] Wang, J., Keightley, P. D., and Halligan, D. L. (2007) Effect of divergence time and recombination rate on molecular evolution of Drosophila INE-1 transposable elements and other candidates for neutrally evolving sites. *J Mol Evol,* **65**(6), 627.

[7] Boeva, V., Popova, T., Bleakley, K., Chiche, P., Cappo, J., Schleiermacher, G., Janoueix-Lerosey, I., Delattre, O., and Barillot, E. (2012) Control-FREEC: a tool for assessing copy number and allelic content using next-generation sequencing data. *Bioinformatics,* **28**(3), 423–425.

[8] Quinlan, A. R. and Hall, I. M. (2010) BEDTools: a flexible suite of utilities for comparing genomic features. *Bioinformatics,* **26**(6), 841–842.

[9] Disdero, E. and Filee, J. (2017) LoRTE: Detecting transposon-induced genomic variants using low coverage PacBio long read sequences. *Mob DNA,* **8**, 5.

[10] Chakraborty, M., Emerson, J. J., Macdonald, S. J., and Long, A. D. (2019) Structural variants exhibit widespread allelic heterogeneity and shape variation in complex traits. *Nat Commun,* **10**(1), 4872.

[11] King, E. G., Merkes, C. M., McNeil, C. L., Hoofer, S. R., Sen, S., Broman, K. W., Long, A. D., and Macdonald, S. J. (2012) Genetic dissection of a model complex trait using the Drosophila Synthetic Population Resource. *Genome Res,* **22**(8), 1558–1566.

[12] Katoh, K. and Standley, D. M. (2013) MAFFT multiple sequence alignment software version 7: improvements in performance and usability. *Mol Biol Evol,* **30**(4), 772–780.

[13] Minh, B. Q., Schmidt, H. A., Chernomor, O., Schrempf, D., Woodhams, M. D., von Haeseler, A., and Lanfear, R. (2020) IQ-TREE 2: New models and efficient methods for phylogenetic Inference in the genomic era. *Molecular Biology and Evolution,* **37**(5), 1530–1534.

[14] Li, H. (2018) Minimap2: pairwise alignment for nucleotide sequences. *Bioinformatics,* **34**(18), 3094–3100.
